# Biomedical Application of Electroactive Polymers in Electrochemical Sensors: A Review

**DOI:** 10.3390/ma12162629

**Published:** 2019-08-18

**Authors:** Damilola Runsewe, Tania Betancourt, Jennifer A. Irvin

**Affiliations:** 1 Materials Science, Engineering and Commercialization Program, Texas State University, San Marcos, TX 78666, USA; 2 Department of Chemistry and Biochemistry, Texas State University, San Marcos, TX 78666, USA

**Keywords:** electroactive polymer, conducting polymer, biosensor

## Abstract

Conducting polymers are of interest due to their unique behavior on exposure to electric fields, which has led to their use in flexible electronics, sensors, and biomaterials. The unique electroactive properties of conducting polymers allow them to be used to prepare biosensors that enable real time, point of care (POC) testing. Potential advantages of these devices include their low cost and low detection limit, ultimately resulting in increased access to treatment. This article presents a review of the characteristics of conducting polymer-based biosensors and the recent advances in their application in the recognition of disease biomarkers.

## 1. Introduction

Conducting polymers (CPs) have found extensive application in the biomedical industry in recent years. This is due to the change in their electrical properties when an electric stimulus is applied [1], which leads to a variety of applications in biosensors [2,3], drug delivery systems [4], tissue engineering scaffolds [5], and energy storage [6]. The focus of this paper is on the application of CPs for use in electrochemical biosensors. CP-based sensors offer advantages, such as the possibilities of flexible medical devices, which can be used in living systems. Additionally, the growing need for on-site diagnosis/detection of acute and chronic disease to enable rapid decision-making has motivated the development of devices that convert disease analyte presence into direct electrical signals in real time.

**Figure 1:**
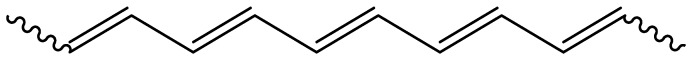
The structure of polyacetylene, showing the backbone containing conjugated double bonds.

Conducting polymers were first synthesized in the 1970s [7]. These materials become conductive upon oxidation (positive doping, or p-doping) or reduction (negative doping, or n-doping) due to conjugation in the polymer chain, characterized by alternating single and multiple (double or triple) bonds, as seen in Figure 1 below [8]. The π electrons of the multiple bonds are delocalized across extended regions of the entire conjugated structure, resulting in polymers that are easily oxidized and/or reduced via a doping process.

**Figure 2:**
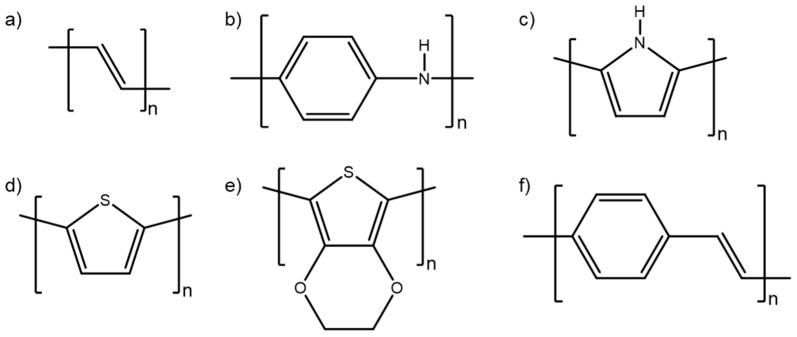
Chemical structures of some common conducting polymers: (**a**) polyacetylene, (**b**) polyaniline (PANI), (**c**) polypyrrole (PPy), (**d**) polythiophene (PT), (**e**) poly(3,4-ethylenedioxythiophene) (PEDOT), and (**f**) poly(phenylene vinylene) (PPV).

Doping of the π-conjugated system causes a reduction in structural and morphological disorder [8], thereby increasing conductivity from the boundary region between insulating materials and semiconductors to the metallic region [9,10], which, in most cases, results in an increase in the conductivity. Conductivities of CPs are typically between 10^−6^ and 10^−10^ S/cm in the neutral state and can reach 10^5^ in the doped state [8]. Figure 2 shows the chemical structures of some common conducting polymers.

Conducting polymers are most commonly prepared via oxidative polymerization (either electrochemical [11] or chemical [12]), or via non-oxidative chemical polymerization methods such as Grignard metathesis [13] or dehydrobrominative polycondensation [14].

## 2. Properties of Conducting Polymers

**Figure 3:**
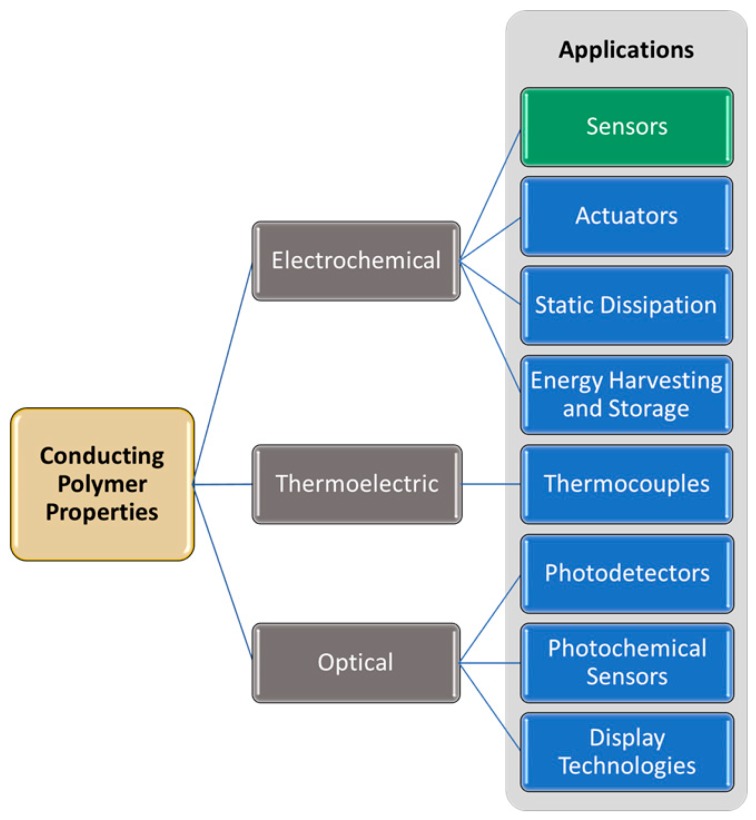
Conducting polymer properties with corresponding applications; the focus of this review is CP-based sensors, highlighted in green.

The unique properties of conducting polymers have brought about research in many areas of fundamental properties and applications, as shown in Figure 3. Along with changes in conductivity, a changing oxidation state also results in changes in other properties, including volume, permeability, reactivity, solubility, optical properties, and thermoelectric properties. There are many excellent reviews covering CP optical properties [15,16], and thermoelectric properties [17] as well as CP applications in energy storage [18,19], photovoltaics [20,21], light emitting devices [22,23], sensors [24,25], and actuators [26,27]. The focus of this review is the use of CPs in electrochemical biomedical sensors.

**Figure 4:**
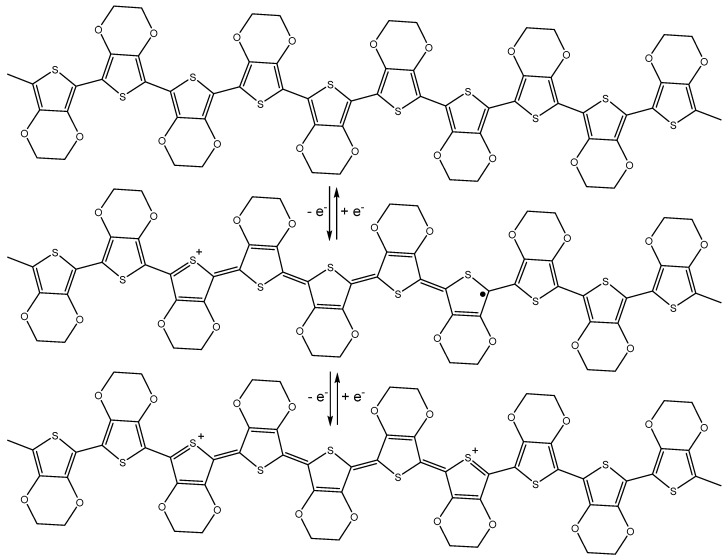
Removal of an electron from neutral PEDOT (top) results in formation of the PEDOT polaron (middle). Removal of a second electron results in the formation of the PEDOT bipolaron (bottom). These processes are reversible.

The interesting electronic properties of CPs arise from their ability to undergo oxidation and reduction reactions. The mechanism of conduction for most heterocyclic polymers, such as PEDOT, involves the removal of electrons in what is known as p-doping (Figure 4), resulting in the formation of a radical cation, also known as a polaron. The radical and cation are typically distributed across three to five quinoidal rings [28]. This is followed by removal of a second electron from the polymer chain, resulting in the formation of a di-cation, also known as a bipolaron [29]. This accounts for the transport properties of CPs: the spinless bipolarons become mobile under an applied electric field, especially at a high doping level, where there is a screening of the coulombic attraction with the counterions, resulting in the easy transport of current [30]. It is important to note that charge transport in CPs requires an electrolyte to provide a source of ions to maintain polymer electroneutrality. Electrolyte choice can significantly impact polymer redox properties, affecting polymer morphology and electrochemical stability, as well as redox potentials [31].

The conductivity of CPs depends on their doping level, which impacts electron density and mobility, as holes (cations) or electrons act as charge carriers [32,33]. Tourillon and Garnier [34] reported the effect of dopants on the electrical properties of CPs. They demonstrated a high interchain conductivity with a corresponding increase in the doping level for thiophene derivatives. As the focus of this review, a survey of the use of electronic properties of CPs as biosensors follows.

## 3. Conducting Polymer-Based Electrochemical Sensors

**Figure 5:**
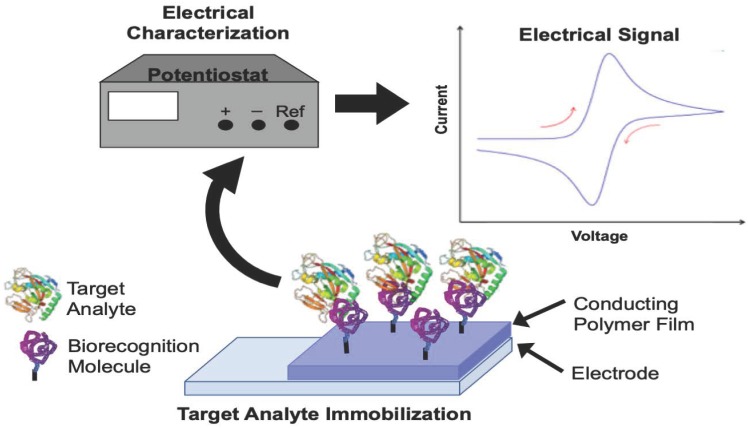
Electrochemical sensing setup.

When CPs are used as electrochemical sensors, an analytical device containing the CP converts the chemical potential of a target analyte into a proportional measurable electrical signal. The main goals of modifying a sensing electrode with a CP are to improve sensitivity and selectivity while reducing the effect of interfering reactions. Figure 5 shows a typical setup of an electrochemical sensor. The CP-modified electrode is attached to a biorecognition molecule, which interacts specifically with the target analyte. Careful choice of the biorecognition molecule allows for improved selectivity. The analyte is interrogated using a variety of electrical characterization methods that result in a measurable signal. Electrochemical sensors can be described from the perspective of the electrochemical characterization methods used to interrogate sensor response (Section 3.1, below) or from the perspective of the type of biorecognition molecules used in the detection approach (Section 3.2, below).

### 3.1. Electrochemical Characterization Methods

A wide variety of electrical methods have been explored for CP-based biosensors, including potentiometry, amperometry, conductometry, and voltammetry. Examples of all these methods follow. 

#### 3.1.1. Potentiometry

In this method, an electrode coated with a CP serves as a sensing membrane that is able to reach a local equilibrium with the species of interest in the solution. The signals of the sensor are a result of the changes in the open-circuit potential of the polymer-modified electrode measured against a suitable reference electrode, which is immersed in the analyte solution.

**Figure 6:**
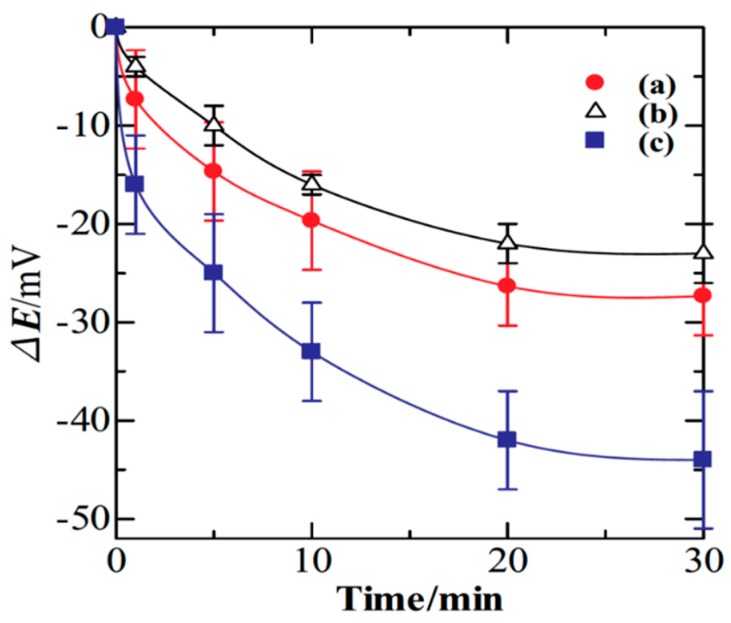
Time dependence of the potential change for a CP/POM electrode in the presence of 0.1 mM solutions of different antioxidants (a–c) [35]. Reprinted from *J. Electroanal. Chem.*, Vol. 828, Y. Tanaka, T. Hasegawa, T. Shimamura, H. Ukeda, and T. Ueda, “Potentiometric evaluation of antioxidant capacity using polyoxometalate-immobilized electrodes,” pp. 102–107, Copyright 2018, with permission from Elsevier.

Tanaka et al. [35] explored the potentiometric evaluation of antioxidant levels found in food using polyoxometalates (POMs). The POMs were electrodeposited on the surface of glassy carbon electrodes (GCE) with one of three CPs: PPy, PANI, or PEDOT. Antioxidant activity is commonly characterized using oxygen radical absorbance capacity (ORAC) and 2,2-diphenyl-1-picrylhydrazyl (DPPH) radical assays, but electrochemical determination of antioxidant activity would be a simple, cost-effective alternative. In Figure 6, change in the potentiometric response (ΔE) of one CP/POM electrode to different antioxidants is monitored over time, with the response leveling off after ~20 min. While the CP/POM electrodes performed similarly to ORAC and DPPH assays, POM was found to slowly release from the electrodes, potentially limiting long-term use. 

**Figure 7:**
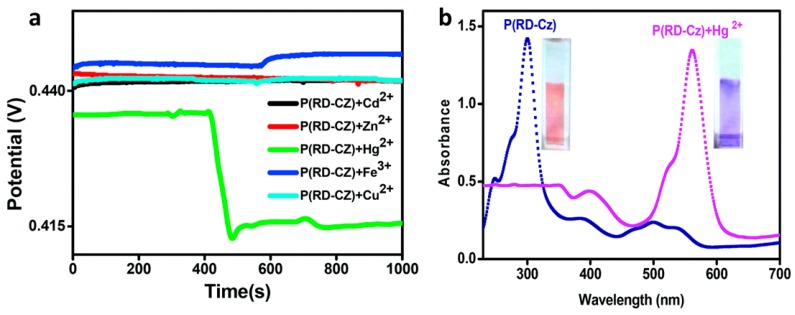
(**a**) A carbazole-based CP electrode’s potentiometric response to Hg^2+^ ions is easily differentiated from its response to other metal ions. (**b**) The same CP can also be used as a colorimetric sensor for Hg^2+^ [36]. Reprinted from *The Analyst*, Vol. 142, R. Ayranci, D. O. Demirkol, S. Timur, and M. Ak, Rhodamine-based conjugated polymers: Potentiometric, colorimetric and voltammetric sensing of mercury ions in aqueous medium,” pp. 3407–3415, Copyright 2017, with permission from the Royal Society of Chemistry.

Ayranci et al. [36] reported a novel way of sensing mercury ions in an aqueous medium by using a rhodamine-functionalized, carbazole-based CP, P(RD-CZ). The carbazole (CZ) group provided the electroactivity, while the rhodamine (RD) group provided fluorescent and colorimetric changes on exposure to Hg^2+^. As can be seen in Figure 7, the sensor exhibited a more pronounced potentiometric response to Hg^2+^ than to other cations, such as Cd^2+^, Cu^2+^, Zn^2+^, and Fe^3+^, suggesting good selectivity. A pronounced colorimetric response can also be used as a rapid, inexpensive, qualitative detection method for Hg^2+^ [36]. 

#### 3.1.2. Amperometry

In this method, an electrochemical signal is derived from changes in current during oxidation and/or reduction, which result from an applied constant voltage that is applied to a polymer-modified electrode. The amperometric method has been widely adopted for use in glucose monitoring [37,38,39]. A wide range of other analytes have also been detected using this method, including dopamine [40], ethanol and methanol [41], and cholesterol [42]. Select examples are given below.

**Figure 8:**
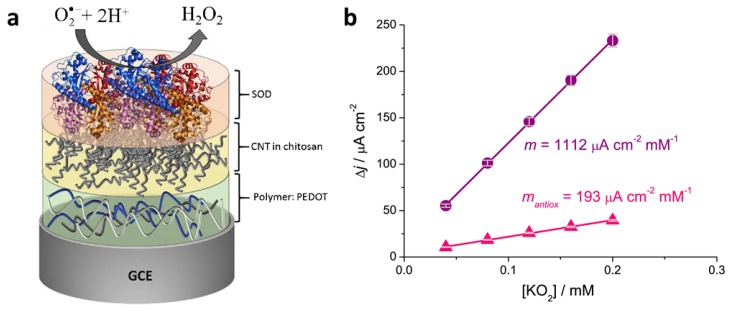
(**a**) Optimal electrode configuration, with PEDOT deposited on a glassy carbon electrode, coated with CNTs in chitosan, which are then coated with superoxide dismutase (SOD). (**b**) Example of determination of relative antioxidant capacity of wine; increasing amounts of KO_2_ are added to the SOD/CNT/PEDOT/GCE sensor in the absence (circles) and presence (triangles) of red wine (8 µL added to 2 mL solution of 0.1 M sodium phosphate buffer containing 0.05 M NaCl (pH 7.0) at an applied potential of −0.3 V vs. Ag/AgCl [43]. Reprinted from *Sensors and Actuators B: Chemical*, Vol. 236, R M. Braik, M. M. Barsan, C. Dridi, M. Ben Ali, and C. M. A. Brett, “Highly sensitive amperometric enzyme biosensor for detection of superoxide based on conducting polymer/CNT modified electrodes and superoxide dismutase,” pp. 574–582, Copyright 2016, with permission from Elsevier.

Braik et al. [43] incorporated multiwalled carbon nanotubes (CNT) with the conducting polymer PEDOT on glassy carbon electrodes in order to detect the reactive oxygen species superoxide, O_2_^•−^, using the enzyme superoxide dismutase (SOD), which converts superoxide radicals to hydrogen peroxide. While sensors prepared without the PEDOT layer were also effective, sensors prepared by layering PEDOT, CNT in chitosan, and SOD exhibited the lowest limit of detection (1 µM), the highest sensitivity (~1115 µA cm^−2^ mM^−1^), and a broad linear range (20–3000 µM). The authors speculated that the chitosan-CNT layer improved performance by providing a biocompatible, porous layer for enzyme immobilization at the electrode surface. This system was applied to determine the antioxidant capacities of several varieties of wine, which contains the antioxidant resveratrol. Figure 8 shows the preferred electrode configuration as well as the use of the sensor to determine the relative antioxidant capacity (RAC) of red wine. The amperometric response of the sensor to the addition of increasing amounts of superoxide (KO_2_) was determined in the absence of and in the presence of red wine. Then, a comparison of the slopes of the plots without and with the wine was used to calculate RAC. 

**Figure 9:**
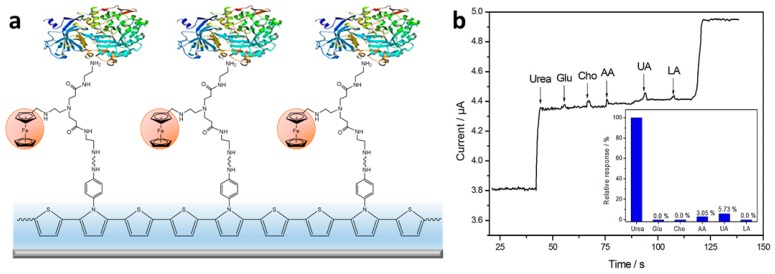
(**a**) Biosensing electrode with immobilized urease. (**b**) Interferents glucose (Glu), cholesterol (Cho), ascorbic acid (AA), uric acid (UA), and lactic acid (LA) have minimal amperometric response relative to urea [44]. Reprinted from *Enzyme and Microbial Technology*, Vol. 102, M. Dervisevic, E. Dervisevic, M. Senel, E. Cevik, H. B. Yildiz, and P. Camurlu, “Construction of ferrocene modified conducting polymer based amperometric urea biosensor,” pp. 53–59, Copyright 2017, with permission from Elsevier.

Subsequently, Dervisevic et al. [44] constructed a ferrocene-modified CP-based sensor for the amperometric determination of urea. A thiophene-pyrrole copolymer with aniline substituents was electropolymerized on a pencil graphite electrode and coated with diaminoferrocene as a mediator (Figure 9a). The enzyme urease was then added via glutaraldehyde crosslinking. The resulting urea biosensor was reported to have a 12 µM detection limit, a linear range of 0.1–8.5 mM, and a sensitivity of 0.54 µA/mM. This biosensor was tested with human blood and urine samples, demonstrating an excellent performance without significant interference from common interferents (Figure 9b).

#### 3.1.3. Conductometry

In this method, the change in electrical conductivity is monitored on exposure to an analyte. This method has been used for decades to analyze conductivity changes in solution due to the exposure of simple electrodes to analytes such as CO_2_ adsorbed from the atmosphere [45] and salt levels in tears [46]. However, in those cases, the technique was fundamentally non-selective, because anything changing the solution conductivity would give a positive response. More recently, the ability to modify electrodes with CPs has allowed this method to be used selectively on a wide variety of analytes including NO_2_ [47], pH [48], and NH_3_ vapor [49]. Specific conductometric biosensor applications of CPs follow.

**Figure 10:**
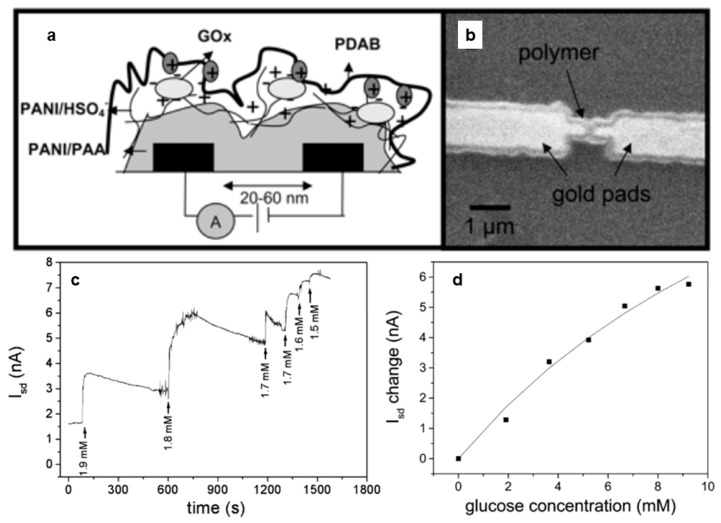
(**a**) Schematic illustration of sensor design. PANI is electrochemically deposited across the 20–60 nm gap between gold electrodes. Then, GOx is immobilized on the PANI surface. (**b**) Scanning electron micrograph of the sensor. (**c**) Drain current changes in response to successive 1 µL additions of 40 mM glucose. E_g_ = 35 mV vs. saturated calomel electrode (SCE) in 20 µL McIlvaine buffer, 0.5 M Na_2_SO_4_ at pH 5. (**d**) Corresponding calibration plot of drain current change vs. glucose concentration [50]. Reprinted with permission from E. S. Forzani, H. Zhang, L. A. Nagahara, I. Amlani, R. Tsui, and N. Tao, “A conducting polymer nanojunction sensor for glucose detection,” *Nano Letters* Vol. 4, pp. 1785–1788, Copyright 2004, American Chemical Society.

Forzani et al. [50] described a conductometric CP sensor for the detection of glucose. The group formed a nanosensor (Figure 10a,b) by coating a pair of nanoelectrodes separated by a small gap (20–60 nm) with PANI/glucose oxidase (GOx). Exposure to glucose resulted in the reduction of GOx. The reduced form of GOx spontaneously reoxidized in the presence of O_2_, producing hydrogen peroxide. The peroxide then oxidized the PANI, resulting in a rapid (<200 ms) local change in conductivity that could easily be measured by monitoring changes in gate current (Figure 10c,d). The authors noted that the spontaneous regeneration of GOx by dissolved oxygen makes these devices ideal for in vivo monitoring of glucose levels.

With the suspected role of dopamine in neuropsychiatric disorders, such as Parkinson’s disease and schizophrenia, methods of in vivo detection of dopamine levels under physiological conditions are needed. Fabre and Taillebois [51] developed interdigitated microarray electrodes coated with poly(aniline boronic acid) to be used as a conductometric sensor for dopamine. As dopamine, an aromatic diol, bonds to the polymer’s immobilized boronic acid groups, the conductivity of the polymer decreases. This is readily detected as a decrease in drain current between the two electrodes at a constant offset potential. The studies were conducted under physiological conditions in a pH 7.4 phosphate buffer solution, demonstrating the potential of this device for in vivo sensing. The device appears to be specific to dopamine; even though ascorbic acid has a similar diol structure, exposure to ascorbic acid does not induce a conductivity change. One potential setback for in vivo use is the need to immerse the dopamine-exposed sensor in sulfuric acid for several minutes to regenerate the sensor. 

**Figure 11:**
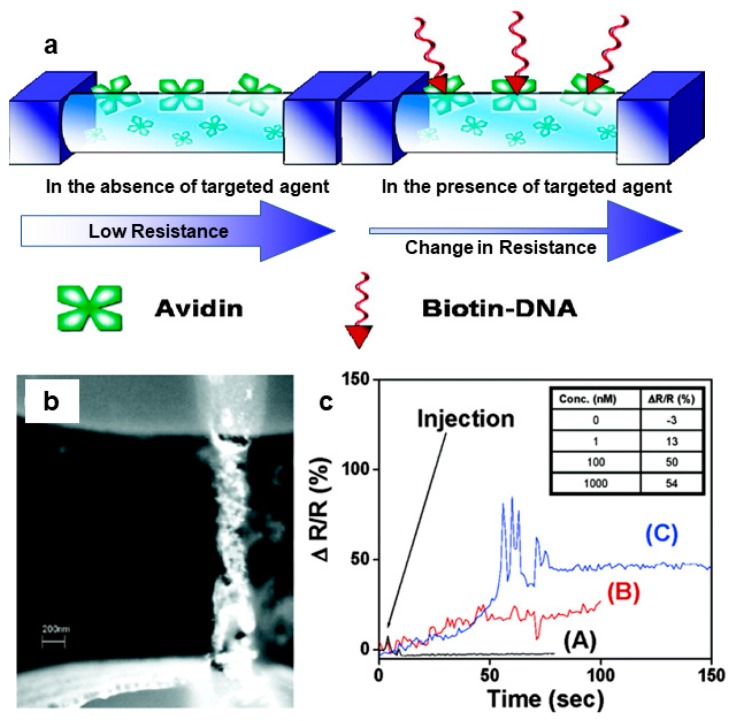
(**a**) Incorporation of some species, such as avidin, enables detection of other species, such as biotin, due to changes in conductivity/resistance. (**b**) Scanning electron micrograph of a 200-nm wide avidin-containing PPy nanowire. (**c**) While a PPy nanowire that does not contain avidin (A) shows no resistance change when biotin is added in 10 mM NaCl, avidin-containing PPy nanowires (B) and (C) exhibit a resistance change when biotin is added [52]. Reprinted with permission from K. Ramanathan, M. A. Bangar, M. Yun, W. Chen, N. V. Myung, and A. Mulchandani, “Bioaffinity sensing using biologically functionalized conducting-polymer nanowire,” *Journal of the American Chemical Society*, Vol. 127, no. 2, pp. 496–497, Copyright 2005, American Chemical Society.

CPs are often modified with biomolecules in order to improve the detection of other biomolecules, as illustrated in Figure 11a. One demonstration of this, reported by Ramanathan et al. [52], involved preparing avidin-containing PPy and PANI nanowires (Figure 11b), then demonstrating that the nanowires could be used to detect biotin conjugated to a 20-mer DNA oligo (biotin-DNA). The nanowire sensors reach saturation of recognition sites by 100 nM biotin-DNA, as indicated by the small (4%) increase in resistance change going from 100 nM to 1000 nM, versus a 37% increase going from 1 to 100 nm.

The electrical conductivity of CPs can be enhanced via the incorporation of metal nanoparticles into conducting polymer [53]. Enhanced conductivity can improve detection limits, as Liu et al. demonstrated when they created a nanocomposite sensor incorporating PEDOT microspheres, platinum nanoparticles, and glucose oxidase. The microstructured morphology led to a highly sensitive glucose sensor exhibiting a linear response from 0.1–10 mM and a detection limit of 1.55 µM [54]. Additionally, the material could be produced using large-scale manufacturing processes, which could eventually result in the ability to mass-produce printable sensors.

#### 3.1.4. Voltammetry

This method obtains an electrical signal by sweeping the electrode potential over a range that is associated with the redox reaction of the analyte. The sensor’s response is derived from the change in the peak current that is associated with the targeted redox reaction. The potential can be scanned using standard wave forms such as cyclic, linear sweep, or square wave voltammetry. This has the advantage of a qualitative (by the location of peaks) and quantitative (through the peak current or area) sensing. Recent work in CP-based voltammetric sensors includes cyclic voltammetric detection of glucose using biohybrid porous PEDOT microparticles loaded with platinum nanoparticles and glucose oxidase [54], square wave voltammetry sensors for detection of dopamine [55], sensor arrays for the discrimination of liquids [56], and differential pulse voltammetry studies of the hormone danazol [57]. Specific examples of voltammetric sensors from recent literature follow. 

**Figure 12:**
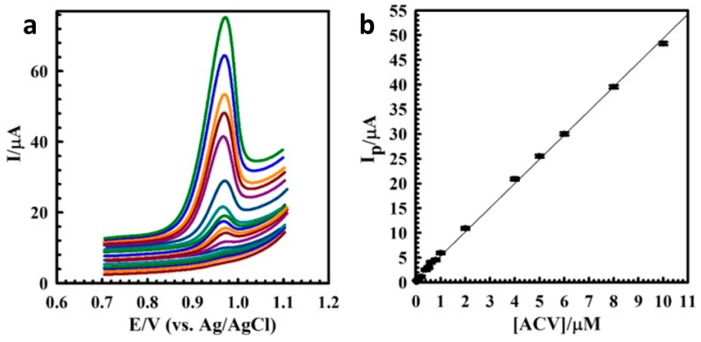
(**a**) Linear sweep voltammograms for various concentrations of ACV in the range of (bottom to top) 0.03–10.00 μM at 100 mV s^−1^ in pH 7.0 phosphate buffer solution after 160 s accumulation time, and (**b**) the corresponding linear calibration curve of peak current vs. ACV concentration [58]. Reprinted from *Materials Science and Engineering C*, Vol. 53, S. Shahrokhian, M. Azimzadeh, and M. K. Amini, “Modification of glassy carbon electrode with a bilayer of multiwalled carbon nanotube/tiron-doped polypyrrole: Application to sensitive voltammetric determination of acyclovir,” pp. 134–141, Copyright 2015, with permission from Elsevier.

The drug acyclovir (ACV) is useful for treating a variety of diseases, including herpes simplex, but high levels can lead to nephrotoxicity and neurotoxicity. Shahrokhian et al. [58] therefore developed a unique and highly selective voltammetric sensor for the determination of ACV. A thin film of multi-walled carbon nanotubes (MWCNTs) was deposited on a GCE and coated with an electropolymerized layer of PPy doped with disodium 4,5-dihydroxy-1,3-benzenedisulfonate (tiron). Linear sweep voltammetry (LSV) was then used to evaluate the electrochemical response of the electrode to ACV. Variables, such as drop size of the cast MWCNT suspension, pH of the supporting electrolyte, amount of PPy, and accumulation time, were optimized by monitoring the LSV response of the modified electrode toward the ACV. They reported the best LSV response at a pH of 7.0 after an open circuit accumulation time of 160 s. A detection limit of 10.0 nM was obtained for ACV, with a linear dynamic range of 0.03–10.0 µM. Figure 12 shows LSVs of solutions containing different concentrations of ACV under the optimum experimental conditions on the surface of the modified electrode [58]. The sensor also proved effective for ACV detection in human serum.

**Figure 13:**
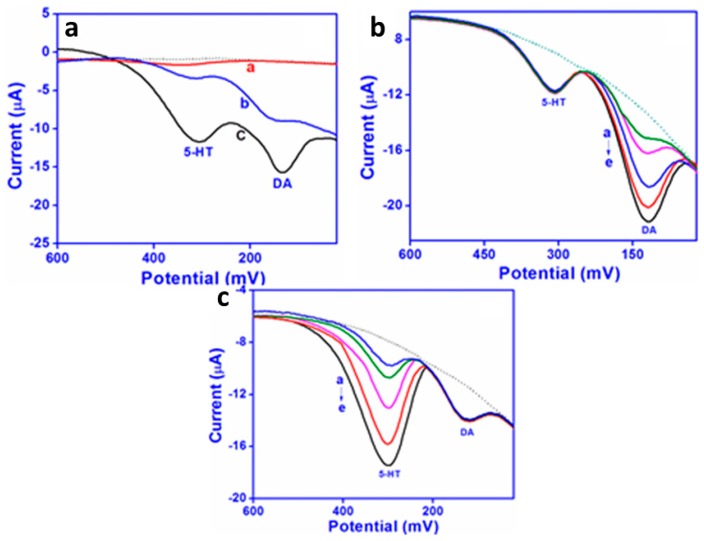
(**a**) A comparison of square wave voltammograms of 50 µM each of dopamine (DA) and serotonin (5-HT) on bare SPC (curve a), graphene-coated SPC (curve b), and CP/graphene-coated SPC (curve c) in phosphate buffer at pH 7.2 at a scan rate of 100 mV s^−1^ using an Ag/AgCl reference electrode. (**b**) Square wave voltammetry in pH 7.2 phosphate buffer with 5-HT concentration held constant at 50 µM while DA concentration was increased from 15 to 80 µM. (**c**) Square wave voltammetry in a pH 7.2 phosphate buffer with the DA concentration held constant at 20 µM while the 5-HT concentration was increased from 5 to 90 µM [60]. Reprinted from *Sensors and Actuators B: Chemical*, Vol 239, M. Raj, P. Gupta, R. N. Goyal, and Y. Shim, “Graphene/conducting polymer nano-composite loaded screen printed carbon sensor for simultaneous determination of dopamine and 5-hydroxytryptamine,” pp. 993–1002, Copyright 2017, with permission from Elsevier.

Simultaneous detection of the two neurotransmitters dopamine and serotonin is desirable because they are both released during the same biological processes [59]. Detection methods currently employed include liquid chromatography, capillary electrophoresis, and mass spectrometry, but these methods require long analysis times and expensive instrumentation. Because dopamine and serotonin are both monoamine-functionalized phenolic derivatives, distinguishing between them electrochemically can be challenging. Raj et al. [60] prepared screen-printed sensors to distinguish between dopamine and serotonin in human biological fluids. The sensors were prepared by drop-casting graphene onto screen-printed carbon electrodes (SPCs) and then electrochemically depositing a polyaniline derivative onto the graphene-coated electrodes. The oxidations of dopamine and serotonin are easily distinguishable voltammetrically (Figure 13a), with a peak at ~180 mV for dopamine and a peak at ~378 mV for serotonin. The improvement in sensitivity for the CP/graphene-coated SPC is readily apparent. Square wave voltammetry was then used to monitor changes in the concentration of dopamine while holding serotonin concentration constant (Figure 13b) and to monitor changes in the concentration of serotonin while holding dopamine concentration constant (Figure 13c). Detection limits were 2 nM in the linear dynamic range 0.05–100 µM for dopamine and 3 nM in the linear dynamic range 0.05–150 µM for serotonin. The method was found to work well in human urine and plasma samples, as well as in pharmacological formulations.

### 3.2. Biorecognition Molecules

Since the first development of biosensors by Updike and Hicks in 1967 [61], molecular recognition has been of major importance to biosensing. Chambers et al. [62] have presented a selective overview of various biorecognition molecules including receptors, enzymes, antibodies, nucleic acids, molecular imprints, and lectins that have impacted biosensor development. In this section, the discussion is restricted to a selective overview of recent developments of some of those types of biorecognition molecules and their application in recent electrochemical CP-based biosensors. The recognition methods discussed are:

- enzyme-based recognition
- antibody-based recognition
- aptamer-based recognition
- peptide-and nucleic acid-based recognition.

#### 3.2.1. Enzyme-Based Recognition

Enzyme biorecognition is of interest due to the range of measurable products that stem from the reaction between enzymes and their substrate, including electrons, protons, heat, chromophores, and light. Urea and glucose are two of the most widely detected analytes with the use of biorecognition elements due to their importance in both environmental and medical settings [63,64,65,66,67,68,69]. 

The working principles for biorecognition molecules like glucose have not changed over the years; when glucose encounters an immobilized enzyme, transduction is usually achieved amperometrically via an electrode. Most of the biosensors for glucose use glucose oxidase (GOx) as their recognition element, which catalyzes the oxidation of glucose to gluconolactone:

Glucose + O_2_ → Gluconolactone + H_2_O_2_

**Figure 14:**
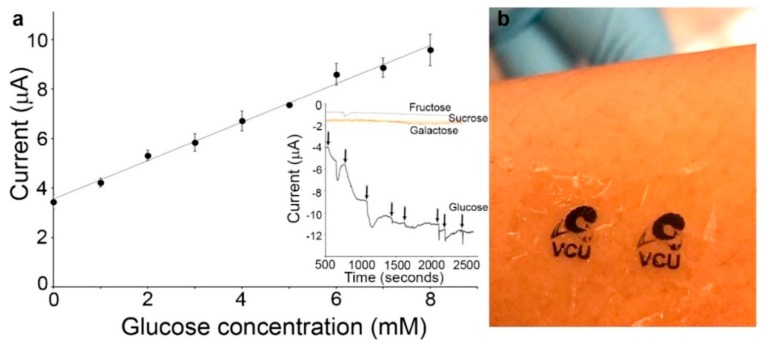
(**a**) SPP-PEDOT:PSS containing immobilized GOx is a selective amperometric sensor for glucose. Response to exposure to other sugars can be seen in the inset. (**b**) Image showing SPP-PEDOT:PSS micropatterned electrodes on the flexible fibroin substrate [70]. Reprinted from *Biosensors and Bioelectronics*, Vol. 81, R. K. Pal, A. A. Farghaly, C. Wang, M. M. Collinson, S. C. Kundu, and V. K. Yadavalli, “Conducting polymer-silk biocomposites for flexible and biodegradable electrochemical sensors,” pp. 294–302, Copyright 2016, with permission from Elsevier.

Pal et al. [70] prepared a novel glucose amperometric biosensor utilizing a CP-based sensing ink. The group created a water-soluble photoresist, SPP, from the silk protein sericin, that had been modified with methacrylate groups. SPP was blended with PEDOT doped with poly(styrene sulfonate) (PEDOT:PSS) to create a photo-curable conductive ink (SPP-PEDOT:PSS). The silk protein fibroin was modified with methacrylate groups and crosslinked to form a transparent, flexible, biodegradable substrate (FPP) on which SPP-PEDOT:PSS was patterned; exposure to UV then crosslinked the ink, creating patterned conductive electrodes. The authors demonstrated that the as-prepared electrodes could be used as non-specific sensors of dopamine and ascorbic acid. By immobilizing glucose oxidase (GOx) in the SPP-PEDOT:PSS composite ink, they were able to create glucose-specific sensors (Figure 14) as well. They obtained a limit of detection and sensor quantification of 1.6 mM and 3.52 mM, respectively, with a sensitivity of 7.57 µA/mM cm^2^.

Liu et al. [54] also fabricated a processable enzyme-hybrid CP nanocomposite for the electrochemical detection of glucose. PEDOT microspheres were prepared via a templating process, and platinum nanoparticles were deposited on the surface prior to the immobilization of GOx. The PEDOT served as a high-surface-area conductive substrate, while the platinum catalyzed the enzymatic process and provided additional selectivity for glucose relative to ascorbic acid and uric acid. The PEDOT nanocomposite sensor exhibited sensitivity of 116 µA mM^−1^ cm^−2^ over a linear range of 0.1–10 mM, 2.7 times better than the best previously reported PEDOT-based glucose sensor [71]. The detection limit was 1.55 µM.

#### 3.2.2. Antibody-Based Recognition

Antibody biorecognition sensors utilize the specificity of antibody–antigen biorecognition interactions. One main advantage of the antibody-based recognition method is that the target analyte, the antigen, does not need to be purified prior to detection. A variety of CP-based electrochemical signal transduction techniques for the detection of different analytes have been developed in recent years that have much lower limits of detection than previously reported. The analytes detected using this method include proteins [72,73,74] and cytokines [75,76,77].

**Figure 15:**
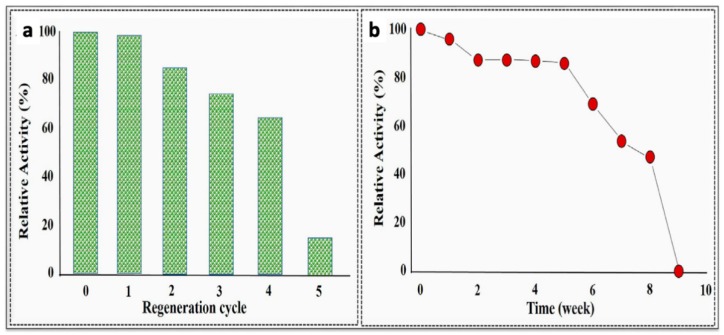
(**a**) The P3-TMA IL-1β sensor could be regenerated with some loss in activity for up to five cycles. (**b**) When stored dry at 4 °C, the sensor retained over 80% of its activity after 5 weeks [78]. Reprinted from *Sensors and Actuators B: Chemistry*, Vol. 270, E. B. Aydın, M. Aydın, and M. K. Sezgintürk, “Highly sensitive electrochemical immunosensor based on polythiophene polymer with densely populated carboxyl groups as immobilization matrix for detection of interleukin 1β in human serum and saliva,” pp. 18–27, Copyright 2018, with permission from Elsevier.

Elevated levels of the cytokine interleukin 1β (IL-1β) are indicative of an immune response to disease or inflammation. Aydin et al. [78] developed a highly selective electrochemical polythiophene-based immunosensor for the detection of IL-1β in human serum and saliva. The researchers prepared a self-assembled monolayer CP-based sensor on indium tin oxide (ITO) to increase the surface area and sensitivity relative to bare ITO. They attached the IL-1β antibody to the malonic acid-functionalized CP, P3-TMA, via amide linkages. Electrochemical impedance spectroscopy was used to measure IL-1β content. A wide linear range of 0.01–3 pg/mL and an exceptionally low detection limit of 3 fg/mL were reported. The sensors could be regenerated with some loss in sensitivity for up to five cycles (Figure 15), and they were stable for five weeks after preparation when refrigerated in their dry state. The authors also provided a summary of other IL-1β immunosensor designs and their performance.

**Figure 16:**
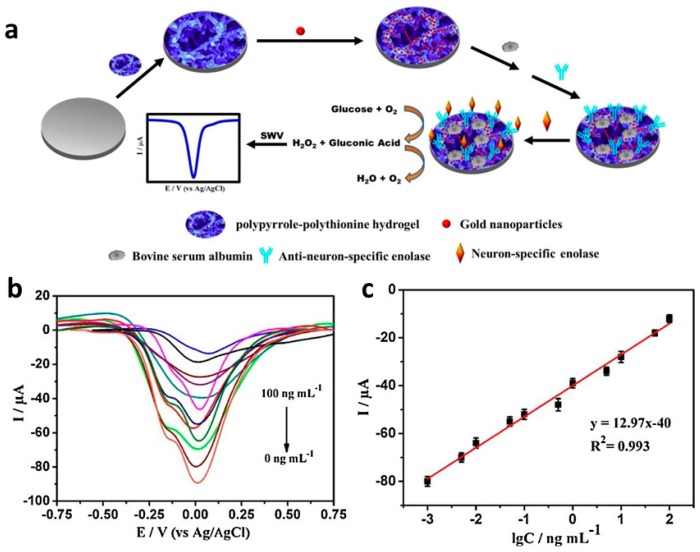
(**a**) A PPy/polythionine hydrogel was prepared containing gold nanoparticles and GOx as the doping agent. (**b**) Square wave voltammograms (SWV) showing the sensor response to NSE at concentrations from 0 ng mL^−1^ to 100 ng mL^−1^ in a 0.1M pH 6.5 phosphate buffer solution with 5 mM glucose. (**c**) Calibration plot with error bars, showing SWV peak current vs. logarithmic values of NSE concentrations [79]. Reprinted from *Sensors and Actuators B: Chemistry*, Vol. 254, H. Wang and Z. Ma, “A cascade reaction signal-amplified amperometric immunosensor platform for ultrasensitive detection of tumour marker,” pp. 642–647, Copyright 2018, with permission from Elsevier.

Identification of tumor markers plays an important role in the early detection of cancers. Current methods of detecting tumor markers are time-consuming and difficult, but electrochemical immunosensors for tumor markers could provide rapid, inexpensive detection. Wang and Ma [79] reported a cascade reaction, signal-amplified, CP-based amperometric immunosensor for the detection of the tumor marker neuron specific enolase (NSE). A glassy carbon electrode surface was coated with a complex hydrogel formulation containing PPy and polythionine along with GOx as a doping agent; gold nanoparticles were generated in situ during the doping process (Figure 16a). The gold nanoparticles enhanced the conductivity and provided a binding surface for the antibody, anti-neuron specific enolase (anti-NSE), and glucose was added to the analyte solution to react with GOx, generating H_2_O_2_ in situ to amplify the signal response. Square wave voltammetry was then used to detect NSE levels (Figure 16b,c). A low detection limit of 0.65 pg mL^−1^ and a wide linear range of 1 pg mL^−1^ to 100 ng mL^−1^ were obtained. Human serum samples were also analyzed, and results were within 10% of those obtained using enzyme-linked immunosorbent assay (ELISA). 

Subsequently, Ma worked with Zhao to develop another immunosensor using the same protocol, replacing PPy with PANI in the hydrogel formula. Instead of using anti-NSE, the researchers introduced anti-CA-125, the antibody for sensing the CA-125 ovarian cancer antigen [80]. Phytic acid was also added as a polymer crosslinker to increase hydrophilicity, providing an antifouling capability. A low detection limit for CA-125 of 0.00125 U mL^−1^ (where 1 U is defined as the amount of the enzyme that catalyzes the conversion of 1 µmole of substrate per minute) was obtained over a wide linear range (0.0001 U mL^−1^–1000 U mL^−1^), which was three times better than had been reported previously. The researchers also found that the immunosensor worked well on human serum samples and was stable for 1 month when stored at 4 °C.

**Figure 17:**
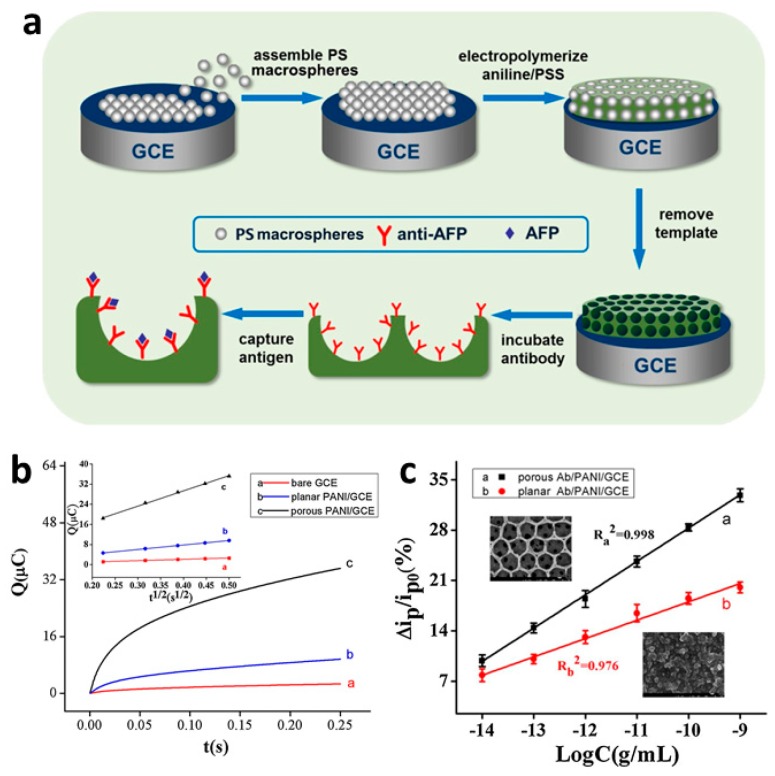
(**a**) Preparation of a microporous PANI:PSS/anti-AFP immunosensor. (**b**) Chronocoulometry of the reduction of 0.2 mM K_3_Fe(CN)_6_ with 0.1 M KCl revealed that the macroporous PANI:PSS (porous PANI/GCE c) electrode exhibited enhanced electroactivity relative to the thin film sensor (planar PANI/GCE b) or a bare glassy carbon electrode (GCE a). Inset shows the relationship between Q and t^½^, which can be used to calculate effective electrode surface area. (**c**) Calibration curves for the AFP detection for porous Ab/PANI/GCE a and planar Ab/PANI/GCE b reveal the enhanced sensitivity of the porous immunosensor for AFP. Insets show scanning electron micrographs of the two electrode materials [82]. Reprinted from *Sensors and Actuators B: Chemistry*, Vol. 255, S. Liu, Y. Ma, M. Cui, and X. Luo, “Enhanced electrochemical biosensing of alpha-fetoprotein based on three-dimensional macroporous conducting polymer polyaniline,” pp. 2568–2574, Copyright 2018, with permission from Elsevier.

Hepatocellular carcinoma can be detected from elevated alpha-fetoprotein (AFP) levels [81]. Liu et al. [82] enhanced the electrochemical immunosensing of alpha-fetoprotein (AFP) by increasing the sensor surface area using macroporous PANI. In their work, polystyrene macrospheres were used as a hard template to prepare a high-surface-area, porous PANI on the surface of a GCE; the PANI was doped with poly(styrene sulfonate) (PSS) to obtain a 3D macroporous PANI:PSS (porous PANI/GCE, Figure 17a). Planar (non-templated) PANI:PSS on a GCE (planar PANI/GCE) was also prepared for comparison. Chronocoulometry (Figure 17b) revealed that the electroactive surface area of the porous PANI/GCE was ≈3.4 times that of the planar PANI/GCE and ≈11.5 times that of bare GCE. The AFP antibody (anti-AFP) was then immobilized on porous PANI/GCE and planar PANI/GCE, and the immunosensing performance of the two electrodes were compared (Figure 17c). Due to its enhanced surface area, the porous sensor (porous Ab/PANI/GCE) exhibited twice the sensitivity of the thin-film, planar sensor (planar Ab/PANI/GCE). The porous Ab/PANI/GCE exhibited a wide linear range of 0.01–1000 pg mL^−1^ and a low detection limit of 3.7 fg mL^−1^. 

#### 3.2.3. Nucleic Acid-Based Recognition

**Figure 18:**
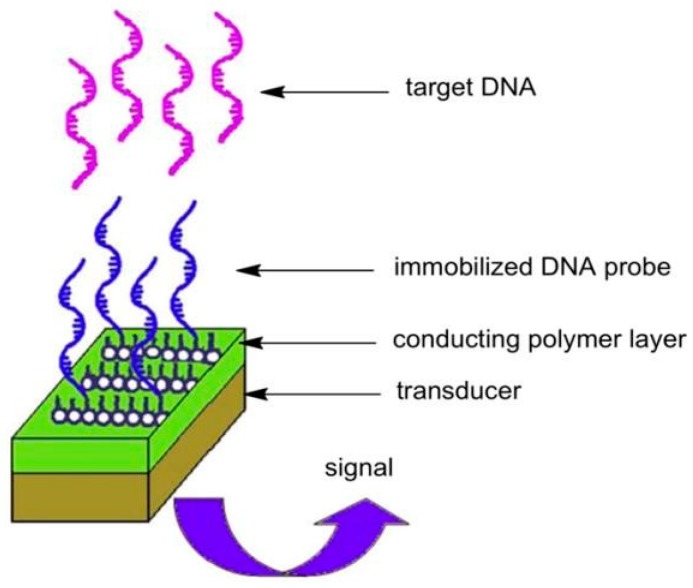
Basic design of a DNA sensor based on a conducting polymer [86]. Reprinted from *Biomaterials*, Vol. 30, H. Peng, L. Zhang, C. Soeller, and J. Travas-Sejdic, “Conducting polymers for electrochemical DNA sensing,” pp. 2132–2148, Copyright 2009, with permission from Elsevier.

There are three major nucleic acid probes used in biosensors: DNA, RNA, and artificially synthesized peptide nucleic acids (PNAs). DNA is preferred due to its improved stability and relative ease of synthesis [83], while RNA aptamers may provide increased binding specificity [84]. These devices rely on the intrinsic electroactivity of DNA [85] and its interaction with an electrochemical or optical sensor. Nucleic acid-based biosensors rely mostly on the immobilization of a single-stranded oligonucleotide probe on a transducing surface in order to recognize a target nucleic acid strand through hybridization as a result of sequence complementarity. The binding of the surface probe to the target single strand is then translated to a useful signal, which can be optical, acoustic, or electrochemical [86]. A typical methodology for binding a nucleic acid to a CP substrate is seen in Figure 18. A single-stranded DNA molecule is immobilized on a CP via entrapment, adsorption, affinity interactions, or covalent bonding. The target DNA is captured via base-pairing, which generates a recognition signal that can be recorded through an electrode. 

Adsorption methods for probe DNA immobilization offer the simplest methodology but may suffer from poor stability and response if not properly optimized. Dutta et al. [87] discussed the use of probe DNA for the detection of a DNA target by using a MoS_2_-PANI nanocomposite. In this work, the probe DNA was immobilized through strong adsorption to the MoS_2_ layer. Methylene blue was utilized as a redox indicator due to its affinity for DNA. A limit of detection of 10^−15^ M and a linear range of 10^−15^ M–10^−6^ M was reported. The linear range obtained was better than what was earlier reported in the literature [88]. The sensor was also reported to be stable (only a 32% decrease in performance) even after 14 days of incubation at 4 °C.

Noncovalent affinity interactions have also been readily used for the immobilization of captured nucleic acid strands as they provide good stability and ease of modification. Shoaie et al. [89] utilized a sandwich hybridization electrochemical biosensor for the detection of target DNA. PANI and gold nanoparticles were utilized to improve charge-carrier transfer. The PANI surface was modified with avidin, and biotinylated DNA capture probes were immobilized utilizing the biotin–avidin noncovalent interactions. A digoxigenin-labeled detector probe was used as the outermost part of the sandwich system to enable interaction with anti-digoxigenin antibody labeled with horseradish peroxidase to provide an amplified electrochemical signal through the enzymatic reaction. Under optimal conditions, a limit of detection of 0.01 fM and a linear range of 0.001–1000 pM were obtained. Similarly, Soni et al. [90] reported an electrochemical sensor based on synthesized PANI nanotubes (PANI-NT) for the detection of a gene associated with chronic myelogenous leukemia (CML). The DNA probe specific to the CML gene was immobilized via avidin–biotin interactions onto the surface of PANI-NTs that were electrophoretically deposited on ITO-coated slides. This work was the first report on the application of template-assisted PANI-NT-based electrodes as a transducing platform for cancer detection. The bioelectrode was used in the detection of both synthetic DNA strands, as well as clinical samples. A limit of detection of 10^−16^ M and a wide linear range of 10^−16^–10^−6^ M was achieved. The limit of detection was better than the 4 × 10^−16^ M previously reported in the literature [91]. 

**Figure 19:**
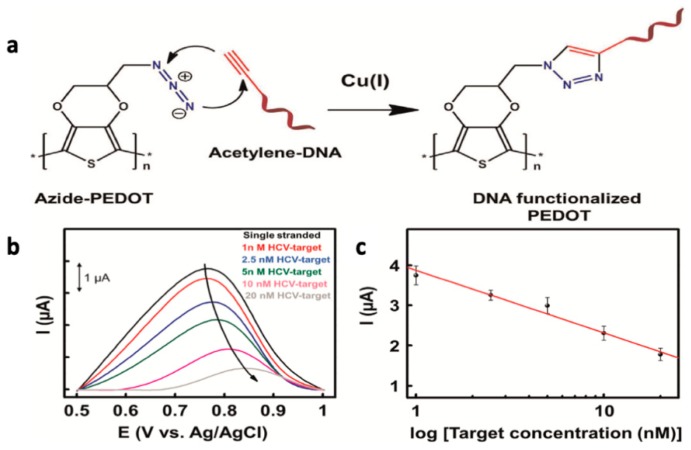
(**a**) DNA immobilization onto azido-PEDOT-modified gold electrodes via click chemistry. (**b**) Amperometric sensor response to increasing concentrations of target DNA, and (**c**) the corresponding calibration curve [92]. Reprinted with permission from *Biosensors and Bioelectronics*, “Label-free electrochemical DNA sensor using “click”-functionalized PEDOT electrodes,” T. Galán, B. Prieto-Simón, M. Alvira, R. Eritja, G. Götz, P. Bäuerle, and J. Samitier, Vol. 74, pp. 751–756, Copyright 2015, with permission from Elsevier.

Covalent DNA immobilization onto the CP surface can be achieved by either polymerizing DNA-functionalized monomers, or by covalently decorating functional CP surfaces with DNA terminated in a suitable terminal group. The former method has a major drawback: the possibility of damaging the oligonucleotide probe from the high potential applied during the polymerization if electrochemical methods are not properly optimized. For demonstration of the post-polymerization immobilization method, Galán et al. [92] reported on the development of a biosensor for the detection of a hepatitis C virus sequence. In their system, acetylene-terminated DNA probes were covalently attached to azido-functionalized PEDOT-modified gold electrodes using copper-mediated click chemistry (Figure 19). The sensor provided a moderate turn-off response with a limit of detection of 0.13 nM of the target DNA sequences. 

Aydemir et al. [93] reported an alternative immobilization method for capturing oligonucleotides. An amine-terminated captured DNA was covalently attached to carboxylic acid-functionalized pyrrole phenylene or thiophene phenylene monomers via carbodiimide chemistry. These monomer-DNA conjugates were then electropolymerized at low potential. Pyrrole or triethylene glycol thiophene phenylene monomers were added to the conjugates to provide control over the density of the DNA-functionalized monomer being polymerized. The researchers probed the sensitivity of these polymers to capture oligonucleotides using electrochemical impedance spectroscopy (EIS). EIS can be used to measure charge transfer resistance and capacitance at interfaces [94], and it may be a more sensitive detection method than cyclic voltammetry [95]. After 50 min of incubation with the complementary oligonucleotides in buffer solutions, EIS revealed remarkable sensitivity, with a limit of detection of 1.6 × 10^−5^ pM for non-Hodgkin lymphoma genes with the CP based on pyrrole phenylene monomers. A limit of detection of 0.4 pM was observed for chronic lymphocytic leukemia (PBGB) genes with the CP based on thiophene phenylene monomers. Both of these are significant improvements over previously reported sensors fabricated using DNA functionalization post-polymerization or DNA entrapment. 

#### 3.2.4. Aptamer-Based Recognition

**Figure 20:**
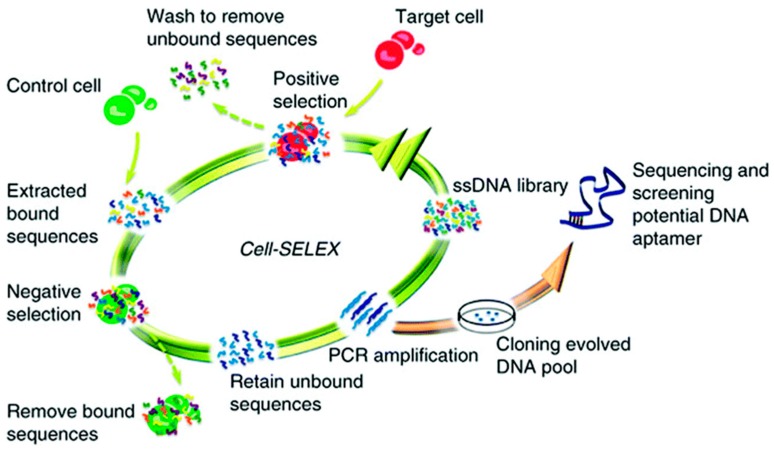
Systematic evolution of ligands using exponential enrichments (SELEX) isolation strategy, applied to RNA aptamer preparation [102]. Reprinted from *Analyst*, Vol. 141, C. Jin, L. Qiu, J. Li, T. Fu, X. Zhang, and W. Tan, “Cancer biomarker discovery using DNA aptamers,” pp. 461–466, Copyright 2016, with permission from the Royal Society of Chemistry.

Aptamers are oligonucleotide (single-stranded DNA (ssDNA) or RNA) strands or peptides that bind to target molecules with high affinity and specificity. Aptamers are gaining favor over the more traditionally used antibodies for biomolecular recognition due to the superior stability of aptamers at elevated temperatures, the relative ease of aptamer preparation and modification using solid-phase synthesis, the low immunogenicity of aptamers, and the ability of aptamers to bind to a wider range of targets, including those that may not elicit an immune response in immunocompetent animals [96]. Ligands utilized in aptamer development have included proteins, organic molecules, peptides, drugs, and even whole cells. Aptamers were first prepared by Gold [97] and Szostak [98] in 1990 using the systematic evolution of ligands using exponential enrichment (SELEX) isolation strategy. In a typical SELEX process, a library of DNA oligonucleotides that contain a portion of random sequence is synthesized. The target analyte is incubated with the nucleic acid pool in a positive selection cycle. Non-binding or low affinity binding nucleic acid molecules are then removed in a wash step, with the captured nucleic acid strands being eluted, recovered, and amplified using PCR [62]. The entire cycle is repeated with the enriched nucleic acid library until a population of high affinity binding nucleic acid strands are isolated, cloned, and sequenced, as shown in Figure 20 for the isolation of an aptamer specific to a set of target cells. Counter, or negative, selection steps can be utilized to select against nucleic acid strands that may have an affinity toward molecularly similar interfering species (control cells in Figure 20). Aptamer-based sensors, also known as aptasensors, have been used for the electrochemical detection of immunoglobulin E [99], fluorescence-based detection of Hepatitis B virus e antigen [100], and colorimetric detection of Vitamin D3 [101]. Examples of CP-based aptasensors are given below.

**Figure 21:**
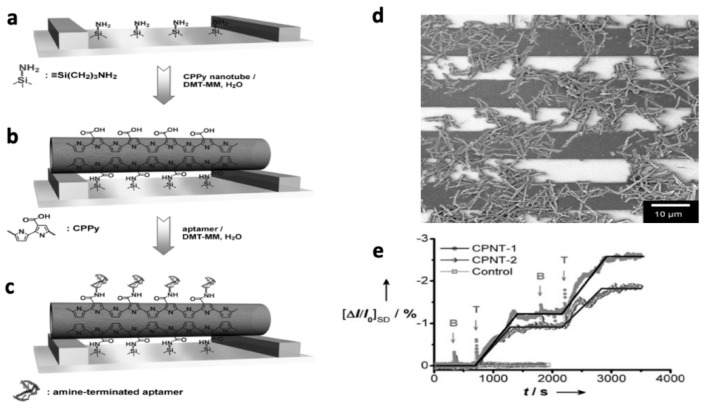
Thrombin sensors are fabricated as shown: (**a**) The interdigitated microelectrodes of the FET gate are treated with aminosilane. (**b**) Immobilization of the CPPy-NTs onto the substrate via amide linkages induced with the condensing agent 4-(4,6-dimethoxy-1,3,5-triazin-2-yl)-4-methylmorpholinium chloride (DMT-MM). (**c**) Thrombin aptamers are bonded to the CPPy-NTs via amide linkages. (**d**) SEM image of CPPy-NTs deposited onto the interdigitated microelectrode substrate. (**e**) Sensing ability of the CPPy-NT FET sensors. Real-time responses of CPPy nanotube FET sensors measured at a source-drain voltage of −15 mV (CPNT-1, circles) and −10 mV (CPNT-2, diamonds): current changes are apparent upon consecutive additions of a 90 nM target (thrombin, T) and nontarget (BSA, B) protein solutions added at times indicated by the arrows. No change in current occurred when the aptamer-free control CPPy-NT FET was used (squares) [104]. Reprinted from *ChemBioChem*, Vol. 9, H. Yoon, J. Kim, N. Lee, B. Kim, and J. Jang, “A Novel Sensor Platform Based on Aptamer-Conjugated Polypyrrole Nanotubes for Label-Free Electrochemical Protein Detection,” pp. 2634–641, Copyright 2008 Wiley-VCH Verlag GmbH & Co. KGaA, Weinheim.

Thrombin is an enzyme that participates in the blood clotting cascade by converting fibrinogen into fibrin; detection of thrombin levels is important to the clinical diagnosis of diseases including leukemia, arterial thrombosis, and liver disease [103]. Its detection is currently accomplished spectroscopically or chromatographically. In an attempt to provide a simpler, rapid alternative to these analyses, Yoon et al. [104] prepared field effect transistors (FETs) utilizing aptamer-functionalized, CP-coated carbon nanotubes (CNTs). The researchers attached carboxylic acid-functionalized PPy (CPPy) to CNTs, immobilized the functionalized nanotubes (CPPy-NTs) on the FET gate, and attached a thrombin aptamer to the polymer via an amide linkage (Figure 21a–d). The aptamer provided selective recognition of thrombin, while the CPPy-NTs provided enhanced charge transport for direct electrochemical detection of thrombin. Non-covalent interactions between thrombin and the aptamer-conjugated polymer chains resulted in a decrease of current flow in the presence of thrombin, as indicated by the negative changes in current response visible in Figure 21 (bottom right). The sensor was specific to thrombin: addition of thrombin resulted in significant changes in current for the FET, while the addition of bovine serum albumin (BSA) had no effect. Additionally, when the FET was tested without the aptamer attached, it was unable to detect thrombin. The CPPy-NT FET sensors were also effective in human blood serum. The researchers did not report detection limits or the linear range for their sensors.

Low levels of the hormone/neurotransmitter epinephrine are observed in patients with Parkinson’s disease and hypertension, while high epinephrine levels may indicate elevated stress or thyroid hormone deficiency [105]. Many methods of detection and other neurotransmitters have been reported (for a review, see Moon et al. [106]). In one example, Shahrokhian et al. [107] prepared a voltammetric epinephrine sensor based on a PPy-carbon nanotube composite. The researchers were able to significantly enhance conductivity via incorporation of the nanotubes and increase the electrode surface area via an over-oxidation procedure, resulting in the ability to control analyte accessibility to the active area of the electrode. This allowed for enhanced detection of epinephrine, even in the presence of the ascorbic acid and uric acid, which are commonly found with epinephrine and have interfered with epinephrine detection in prior methods. The group reported a wide linear range (0.1–100 µM) and a low detection limit of 0.04 µM.

**Figure 22:**
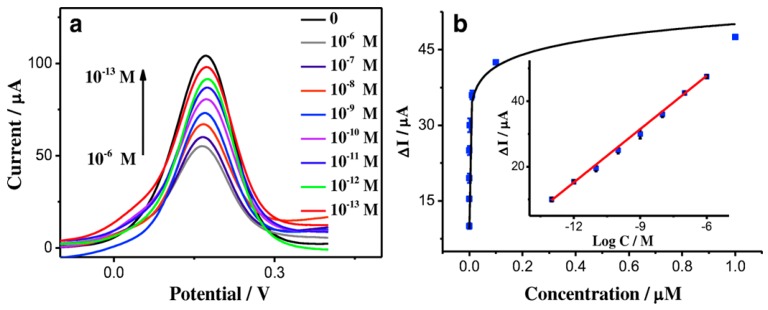
(**a**) Differential pulse voltammetry (DPV) response of the aptasensor to a series of ATP solutions with concentrations from 10^−13^ to 10^−6^ M at a working voltage of 0.18 V vs. SCE. The current response decreases as ATP concentration increases due decreased ionic content at the electrode surface. (**b**) The corresponding response plot and (inset) calibration plot for the aptasensor (R^2^ = 0.998) [109]. Reprinted with permission from Springer *Microchimica Acta*, “A glassy carbon electrode modified with graphene oxide, poly(3,4-ethylenedioxythiophene), an antifouling peptide and an aptamer for ultrasensitive detection of adenosine triphosphate,” Z. Li, J. Yin, C. Gao, L. Sheng, and A. Meng, Vol. 186, Copyright 2019.

Adenosine triphosphate (ATP) is the main energy-releasing component of cells. Abnormal ATP levels may cause diseases such as Alzheimer’s and Parkinson’s [108]. While there are many spectroscopic and electrochemical methods of ATP detection, enhanced detection limits and less-expensive fabrication methods are needed to make electrochemical sensing of ATP viable. Li et al. [109] fabricated a CP-based aptasensor based on an electrochemically deposited graphene oxide-PEDOT nanocomposite on a GCE. A zwitterionic peptide was bonded to the surface to prevent surface fouling by other competing biomolecules. Finally, the ATP binding aptamer, was bonded to the surface to provide specificity for ATP. Human serum albumin was used to test non-specific binding, and minimal change in the current response was observed, The resulting voltammetric sensor exhibited a low detection limit (0.03 pM), a wide linear range (0.1 pM–1.0 µM), and a sensitivity of 2675 µA µM^−1^ cm^−2^ (Figure 22), which were significant improvements over values previously reported in the literature [110].

Ampicillin and kanamycin are small-molecule antibiotics used for the treatment of a range of bacterial infections. Daprà et al. [111] developed an aptamer-based biosensor for the detection of these antibiotics utilizing two independent DNA aptamers specific to these molecules. A polymeric microfluidic chip was used to encase interdigitated electrodes of tosylate-doped PEDOT and poly(hydroxymethyl-EDOT). The two aptamers were covalently attached to the electrodes via amide linkages. This biosensor detected ampicillin in the range of 100 pM to 1 μM and kanamycin from 10 nm to 1 mM with the use of electrochemical impedance spectroscopy. Importantly, the authors demonstrated the detection of ampicillin in milk at a concentration significantly below the maximum residue limit permitted by the regulations of the European Union. 

**Figure 23:**
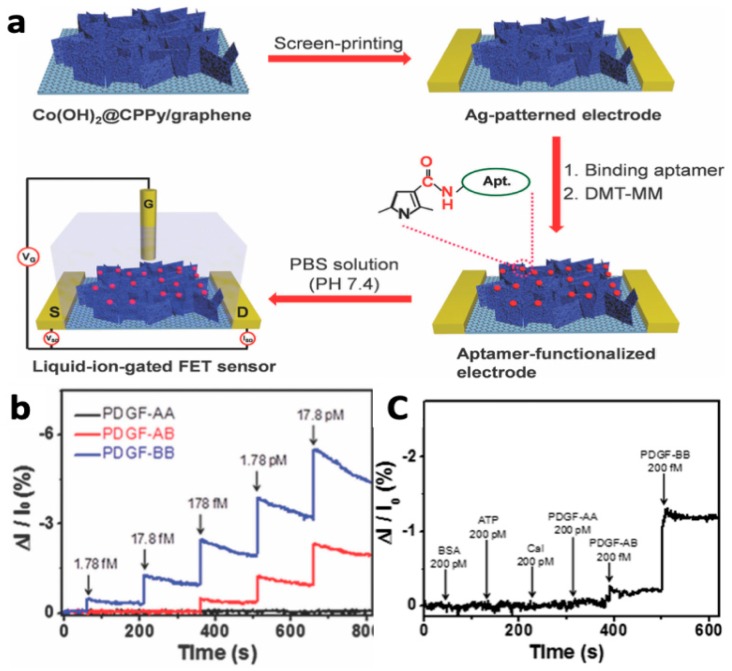
(**a**) Fabrication of a FET sensor. (**b**) Normalized current change response to varying concentrations of three forms of PDGF or (**c**) equal concentrations of PDGF-BB or a series of interfering agents, demonstrating high sensitivity and selectivity for PDGF-BB [112]. Reprinted with permission from Springer *Journal of Materials Chemistry B*, “Multidimensional hybrid conductive nanoplate- based aptasensor for platelet-derived growth factor detection,” J. S. Lee, W. Kim, S. Cho, J. Jun, K. H. Cho and J. Jang, Vol. 4, pp. 4447–4454, Copyright 2016.

Platelet derived growth factor (PDGF-BB) is a pro-angiogenic protein that is up-regulated in response to cancer cell hypoxia, regulating cell growth and division. As such, PDGF is a biomarker of a range of cancers and its levels are associated with cancer progression and reduced survival. Lee et al. developed a multi-dimensional hybrid pyrrole-based FET sensor for the detection of PDGF-BB [112]. Specifically, a multi-layer system containing a poly(ethylene naphthalene), graphene, cobalt hydroxide nanosheets, and 3-carboxylated PPy (Co(OH)_2_@CPPy/graphene) was screen printed with silver paste to form the FET setup and was covalently functionalized with a DNA aptamer specific for PDGF-BB (Figure 23a). Amperometric measurements demonstrated that the high surface area provided by the conductive polymer-functionalized cobalt hydroxide nanosheets provided ultra-high sensitivity (1.78 fM), high selectivity relative to other forms of PDGF and other interfering agents, and a 4-week lifetime when stored at room temperature (Figure 23b). The sensitivity demonstrated was 10–100 times higher than that of other reported FET sensors. 

#### 3.2.5. Biosensors Based on Other Recognition Molecules

In addition to the discussed biomolecules of detection reported earlier, other non-enzymatic, non-nucleic acid, non-antibody-based recognition molecules have been made specifically for particular analyte detection. In some cases, CP-modified electrodes and CP-based composites have been used directly without the need for a biorecognition molecule. These systems, however, may suffer from low selectivity. In other cases, recognition molecules, such as polysaccharides or molecularly imprinted polymers, are utilized to provide selective recognition toward given analytes. Examples of both types of systems are discussed below.

Hamid et al. fabricated a PANI based sensor for the detection of Danazol (Dz), which was characterized using DPV and electrochemical impedance spectroscopy [57]. Dz is a synthetic steroid used for the treatment of endometriosis, certain breast diseases, and hereditary angioedema. PANI was electrochemically deposited onto carbon electrodes utilizing HCl as a dopant. Direct interaction of the biosensor with solutions of Dz resulted in a dose-dependent reduction in the current peak when measured using DPV and an increase in the charge transfer resistance when measured via electrochemical impedance spectroscopy. The authors attributed the sensing capability of this biosensor to the coupling of Dz and chloride ions (dopant), which decrease the permeability of these ions during the PANI redox process. This work demonstrated the detection of 2 nM of Dz [57] but did not evaluate the selectivity of the sensor. 

Metal oxides have also been used as biosensing materials in conjunction with CPs. Fayemi et al. [55] utilized transition metal oxides (NiO, ZnO, and Fe_2_O_4_) that were integrated with PANI for dopamine detection via the oxidation of dopamine. The PANI-MO nanocomposite was prepared by mixing MO nanoparticles and PANI in DMF and drop casting the mixture onto bare GCE. PANI-NiO was reported to be the best metal oxide-PANI-modified electrode out of the three nanocomposites tested with a limit of detection of 15.3 nM and a linear response range from at least 0.2 to 2.4 µM. This is within the limit of neurotransmitters in biological fluids [113]. 

Another dopamine-detecting biosensor based on PEDOT was fabricated by Xu et al. [114]. The authors came up with an innovative way of detecting dopamine using PEDOT-modified laser-scribed graphene (LSG). LSG electrodes were fabricated on polyimide films. When PEDOT was then electrodeposited on the LSG film, the PEDOT structurally incorporated into the LSG matrix due to the strong interaction between the LSG and carbon materials, as reported by Taylor et al. [115]. The device had a 0.33 µM limit of detection over a linear range of 1–150 µM and a sensitivity of 0.220 ± 0.11 µA/µM. Notably, the sensor performed well in the presence of uric acid and ascorbic acid, known interfering agents for dopamine.

Li et al. [116] utilized a glassy carbon electrode (GCE) modified with PEDOT and gold-graphene core-shell nanoparticles for the detection of the drug paracetamol. Paracetamol is an inherently electroactive molecule; however, its detection was significantly enhanced in GCE-modified PEDOT/gold-graphene nanocomposites when compared to PEDOT-modified electrodes or bare GCE. A limit of detection of 41 nM and a linear response range 0.15 µM–5.88 mM were demonstrated with the composite system.

Mandal et al. [117] reported on the development of an electrochemical biosensor for the detection of the α-amylase enzyme in human serum based on a PANI-emeraldine salt film grown on a glass substrate that was populated with starch-coated gold nanoparticles (SAuNps). α-Amylase interaction with the composite biosensor catalyzed the hydrolysis of starch stabilized on the gold NPs, thereby causing changes in the electrical resistance of the sensor that were proportional to the concentration of the enzyme. Sensitive detection in the linear range of 25–100 U/L was demonstrated, which is satisfactorily within the normal level of the α-amylase in blood serum [118]. 

**Figure 24:**
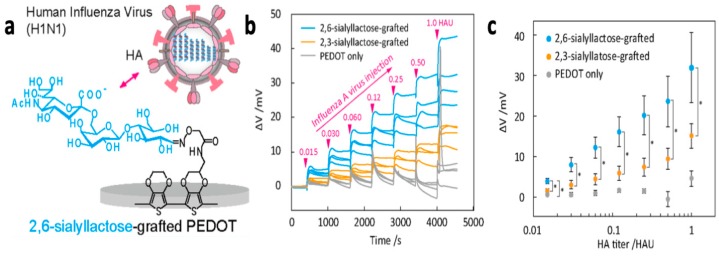
(**a**) Design of siallyllactose-based PEDOT sensor for detection of H1N1 virus. (**b**) Potential changes as a function of time upon the sequential addition of H1N1 virus solutions of varying concentrations to PEDOT-only (control) or siallyllactose-granfted PEDOT. HAU—hemagglutinin units. (**c**) Potential changes as a function of H1N1 virus concentration. * *p* < 0.05 [119]. Reprinted with permission from *ACS Applied Materials and Interfaces*, “Specific Recognition of Human Influenza Virus with PEDOT Bearing Sialic Acid-Terminated Trisaccharides,” W. Hai, T. Goda, H. Takeuchi, S. Yamaoka, Y. Horiguchi, A. Matsumoto and Y. Miyahara, Vol. 9, pp. 14162–14170, Copyright 2017 with permission from the American Chemical Society.

Hai et al. [119] developed a PEDOT-based sensor for the detection of human influenza A virus (H1N1). This sensor was prepared using electrochemical co-polymerization of EDOT and oxylamine functionalized EDOT, followed by covalent decoration with sialyllactose trisaccharides, which interact specifically with hemagglutinin on the envelope of the H1N1 virus (Figure 24a). Potentiometric studies demonstrated dose-dependent increase in open circuit potential for the sialyllactose-grafted biosensors and no response for the PEDOT-only system (Figure 24b,c). Further studies confirmed the specificity of the siallyllactose-PEDOT sensor toward H1N1 compared to H5N1 virus hemagglutinin. A level of detection of 0.013 hemagglutinin units was achieved, which is two orders of magnitude better than commercial immunochromatographic tests, and comparable to other systems in development, including surface plasmon resonance and ion-sensitive field-effect transistors. 

**Figure 25:**
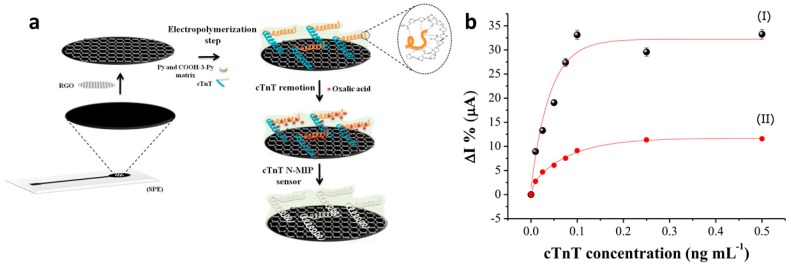
(**a**) Preparation of conductive MIP-based biosensor for the detection of cTnT via the electropolymerization of pyrrole and carboxylated pyrrole in the presence of cTnT, followed by cTnT removal using oxalic acid. (**b**) Response of an MIP sensor (black dots, I) versus a non-imprinted sensor (prepared in the absence of cTnT), demonstrating enhanced sensitivity for MIP systems [120]. Reprinted with permission from *Biosensors and Bioelectronics*, “An ultrasensitive human cardiac troponin T graphene screen-printed electrode based on electropolymerized-molecularly imprinted conducting polymer,” B. V. M. Silva, B. A. G. Rodríguez, G. F. Sales, M. D. P. T. Sotomayor, and R. F. Dutra, Vol. 77, pp. 978–985, Copyright 2016 with permission from Elsevier.

Human cardiac troponin T (cTnT) is a biomarker used to diagnose an acute myocardial infarction, also known as a heart attack. Silva and colleagues [120] developed a biosensor for the detection of TnT utilizing screen-printed, reduced graphene oxide-modified electrodes decorated with a conductive molecularly imprinted polymer (MIP). MIPs are biomimetic receptors that are synthetically prepared by polymerizing monomers in the presence of the target analyte (template). Upon template removal, this process generates a three-dimensional polymer matrix that provides cavities (biomimetic receptors) with the correct size, shape, and electrostatic environment to specifically interact with the molecular target. In this work, a biosensor was generated via the electropolymerization of pyrrole and carboxylated pyrrole (1:5 molar ratio) in the presence of cTnT, as shown in Figure 25. The authors demonstrated a very low limit of detection (0.006 ng/mL) and a linear range of 0.01–0.1 ng/mL. In addition, the sensor performed well in diluted human serum samples, demonstrating selectivity toward cTnT. This biomimetic sensor thereby offers good performance and selectivity with the added advantage of utilizing a low-cost and stable biorecognition mechanism relative to the use of enzymes, antibodies, aptamers, or other biomolecules. 

## 4. Conclusions

Many conducting polymers have been developed and used in the fabrication of electrochemical sensors. The recent work gives an insight into the more promising approaches that are currently available. Conductive polymers offer significant chemical versatility, including a variety of backbone compositions with distinct electrochemical behavior that can be addressed through a range of techniques including potentiometry, amperometry, conductometry, and voltammetry. Also, conductive polymers can include functional groups that enable functionalization with various types of biorecognition molecules. These properties, in addition to their ability to act in conjunction with organic/inorganic materials to provide high sensitivity and selectivity, make them prime substrates for the detection of biological analytes, as described in the examples noted in this review. 

The versatility of conductive polymer biosensors has opened doors to their use in a range of bioanalytical applications including in disposable personal monitoring devices for diagnostic purposes (glucose, fertility, cholesterol, and genetic sensors), point of care diagnostics for clinical use (detection of infectious disease, cardiovascular diseases, and cancer), implantable devices for medical monitoring (neural probes and stimuli-responsive drug delivery systems), and even tissue engineering scaffolds. Their adoption by patients and clinicians could potentially revolutionize current medical practice and the healthcare system by shifting the focus from traditional off-site laboratory analysis to on-the-spot detection and quantification. In addition, the adoption of conductive polymer-based biosensors by the healthcare system could be significant for the early detection of serious diseases such as cancer through biomarker-based screening. Similarly, rapid biomarker detection could be critical for proper selection of personalized treatment options that are suitable for the molecular fingerprint of the patient’s disease (i.e., personalized medicine), as well as for monitoring treatment effectiveness and disease recurrence during and after treatment. 

While advances have been made in the use of conducting polymers for the fabrication of highly sensitive biosensors, one problem that needs to be explored is the long-term stability of these sensors. Current and future work includes sensor miniaturization and multiplexing to enable the development of lab-on-a-chip devices, portable, and even wearable multiplex sensors that can monitor multiple analytes. While the use of conductive polymer biosensors in external or ex vivo devices only requires compatibility with biological fluids, implantable systems for in vivo use must be biocompatible. While the biocompatibility of many conductive polymers has been demonstrated in vitro with a range of cell lines and in animal models, their biocompatibility can be improved by decorating them with biocompatible molecules or blending with other biocompatible materials. Readers are referred to reviews by Balint [121] and Tomczykowa [122] for more information on the biocompatibility and stability of conductive polymers being used in biomedical applications.
